# A Novel Mouse Model of iNKT Cell-deficiency Generated by CRISPR/Cas9 Reveals a Pathogenic Role of iNKT Cells in Metabolic Disease

**DOI:** 10.1038/s41598-017-12475-4

**Published:** 2017-10-06

**Authors:** Yue Ren, Etsuko Sekine-Kondo, Risa Shibata, Megumi Kato-Itoh, Ayumi Umino, Ayaka Yanagida, Masashi Satoh, Komaki Inoue, Tomoyuki Yamaguchi, Keiichi Mochida, Susumu Nakae, Luc Van Kaer, Kazuya Iwabuchi, Hiromitsu Nakauchi, Hiroshi Watarai

**Affiliations:** 1 Division of Stem Cell Cellomics, Center for Stem Cell Biology and Regenerative Medicine, Institute of Medical Science, University of Tokyo, Minato-ku, Tokyo, Japan; 2 The Neurological Institute of Jiangxi Province, Department of Neurology, Jiangxi Provincial People’s Hospital, Nanchang, Jiangxi China; 3 Laboratory of Systems Biology, Center for Experimental Medicine and Systems Biology, Institute of Medical Science, University of Tokyo, Tokyo, Japan; 4 Division of Stem Cell Therapy, Center for Stem Cell Biology and Regenerative Medicine, Institute of Medical Science, University of Tokyo, Tokyo, Japan; 5 Department of Immunology, Kitasato University School of Medicine, Sagamihara, Japan; 6 Cellulose Production Research Team, RIKEN Center for Sustainable Resource Science, Yokohama, Japan; 7 Department of Pathology, Microbiology and Immunology, Vanderbilt University School of Medicine, Nashville, Tennessee USA; 8 Institute for Stem Cell Biology and Regenerative Medicine, Department of Genetics, Stanford University School of Medicine, Stanford, California, USA; 9 Present Address: Wellcome Trust-Medical Research Council Cambridge Stem Cell Institute, University of Cambridge, Cambridge, CB2 1QR UK

## Abstract

iNKT cells play important roles in immune regulation by bridging the innate and acquired immune systems. The functions of iNKT cells have been investigated in mice lacking the *Traj18* gene segment that were generated by traditional embryonic stem cell technology, but these animals contain a biased T cell receptor (TCR) repertoire that might affect immune responses. To circumvent this confounding factor, we have generated a new strain of iNKT cell-deficient mice by deleting the *Traj18* locus using CRISPR/Cas9 technology, and these animals contain an unbiased TCR repertoire. We employed these mice to investigate the contribution of iNKT cells to metabolic disease and found a pathogenic role of these cells in obesity-associated insulin-resistance. The new *Traj18*-deficient mouse strain will assist in studies of iNKT cell biology.

## Introduction

Invariant natural killer T (iNKT) cells are characterized by their expression of a semi-invariant T cell receptor (TCR), Vα14 Jα18 (*Trav11-Traj18)* paired with Vβ8.2 (*Trbv13-2*), Vβ7 (*Trbv29*), or Vβ2 (*Trbv1*) in mice and Vα24 Jα18/Vβ11 (*TRAV10-TRAJ18*/*TRBV25-1*) in humans. These TCRs recognize glycolipid antigens such as α-galactosylceramide (α-GalCer) presented by the monomorphic major histocompatibility complex (MHC) class I-like molecule, CD1d. iNKT cells are potent immune regulators due to their rapid and massive production of a wide range of cytokines upon stimulation, such as IFN-γ, GM-CSF, IL-4, IL-13, IL-17A, and IL-10^1,2^. This feature enables iNKT cells to bridge the innate and acquired immune systems, and to participate in immune responses during a variety of conditions, including infection, autoimmunity, allergy, tumorigenesis, as well as obesity and metabolic disease^3–5^.

NKT cell-deficient mouse strains, including *Traj18*-deficient (*Traj18* ^−/−^) mice that selectively lack iNKT cells, and CD1d-deficient (*Cd1d* ^−/−^) mice that lack both iNKT (also called type 1 NKT) cells and variant NKT (vNKT or type 2 NKT) cells, have greatly facilitated studies on NKT cells. Investigators have employed *Traj18* ^−/−^ mice to study iNKT cell functions, and have compared immune responses in *Traj18* ^−/−^ and *Cd1d* ^−/−^ mice to investigate type 2 NKT cell functions. However, the *Traj18* ^−/−^ mouse strain that was generated using traditional embryonic stem cell/gene targeting technology and has been widely used in iNKT cell studies^6^, was reported to contain an impaired TCR repertoire diversity, due to the *PGK-Neo* ^r^ cassette that was employed to replace the *Traj18* allele and might have inadvertently caused alterations in TCR gene transcription and rearrangement^7^. These findings therefore call into question prior studies that have employed these *Traj18* ^−/−^ animals to reach conclusions about iNKT cell functions.

iNKT cells are enriched in human and murine adipose tissue^8^. However, functional analyses from different research groups using models of NKT cell-deficiency (*Cd1d* ^−/−^or *Traj18* ^−/−^ mice) have reached divergent conclusions^9–11^. It is possible that the lower TCR diversity of the *Traj18* ^−/−^ mice used in some of the studies could potentially contribute to these divergent results.

Recently, three new *Traj18* ^−/−^ mouse lines have been established by different research groups. Two lines were generated by Cre/lox technology^12,13^, and a third was generated by transcription activator-like effector nuclease (TALEN) methodology^14^. Each of these *Traj18* ^−/−^ mouse lines was shown to contain a selective deletion of the *Traj18* locus and iNKT cell-deficiency, in the absence of a biased TCR repertoire. Concerning iNKT cell functions, these animals have thus far been employed to investigate the role of iNKT cells in allergen-induced pulmonary inflammation^12^ and α-GalCer-mediated suppression of tumor metastases^13^. Additional studies are needed to reassess iNKT cell functions and those of type 2 NKT cells with these novel *Traj18* ^−/−^ mouse lines and *Cd1d* ^−/−^ mice.

Here we have successfully generated new *Traj18* ^−/−^ mouse lines by using the Clustered Regularly Interspaced Short Palindromic Repeat (CRISPR)/Cas9 system. Both FACS analysis and cytokine production results confirmed the lack of iNKT cells in the newly generated *Traj18* ^−/−^ mouse strains in both C57BL/6 J (B6) and BALB/cAJcl (BALB/c) backgrounds. Analysis of the TCRα repertoire confirmed that these *Traj18* ^−/−^ mice harbor an undisturbed TCRα repertoire. Using this new mouse strain on the B6 background, we re-assessed the contribution of iNKT cells to obesity-associated metabolic disease, and found that obese *Traj18* ^−/−^ mice show reduced weight gain and ameliorated metabolic parameters, thus indicating a pathological role of iNKT cells in the development of obesity-associated disorders.

## Results

### Design of a single guide RNA for *Traj18* locus mutation

Two *Traj18* gene-targeted single guide RNAs (called Traj18_sgRNA1 and Traj18_sgRNA2) (Supplemental Fig. 1a) were designed to target the *Traj18* gene segment. We first validated whether the sgRNAs could recognize and cleave the *Traj18* target sequence using an *in vitro* system, as described previously^15^. In brief, the targeted genome segment of the *Traj18* locus (Supplemental Fig. 1b), including sgRNA target sequence, was inserted between the split-EGFP (enhanced green fluorescent protein) fragments that share 400 bp of DNA sequence, under control of the CAG promoter (pCAG-EGxnFP-target) and used as a reporter plasmid. We co-transfected pCAG-EGxnFP-target and pCAG-T3-hCas9-pA with or without pU6-sgRNA (Supplemental Fig. 1c) into HEK293T cells and the levels of reconstituted EGFP expression were evaluated by fluorescence microscopy (Supplemental Fig. 1d) and flow cytometry (Supplemental Fig. 1e) 48 hrs after transfection. Both Traj18_sgRNA1 and Traj18_sgRNA2 worked effectively, as revealed by EGFP expression in approximately 40% of the transfected cells.

### Generation of mice with a partial deletion of the *Traj18* gene segment by CRISPR/Cas9 technology

Following validation of sgRNAs in HEK293T cells, we proceeded to generate *Traj18* gene-targeted mutant mice by zygote injection. sgRNA and hCas9 mRNA were placed under the phage T3 promoter followed by *in vitro* transcription using T3 RNA polymerase (Supplemental Fig. 2a) and injected into the pronuclei of fertilized eggs of B6 mice. Pups derived from these fertilized eggs were genotyped by sequence analysis. Eight out of 11 mice from the Traj18_sgRNA1 (Supplemental Fig. 2b, Supplemental Table 1) and 10 out of 17 mice from the Traj18_sgRNA2 (Supplemental Fig. 2c, Supplemental Table 1) contained a partial deletion in the *Traj18* locus. We selected three founder mice and established four new strains with a *Traj18*-partial deletion, *Traj18* ^−/−^ (1-1 L), *Traj18* ^−/−^ (1-1 S) and *Traj18* ^−/−^ (1-2) derived from Traj18_sgRNA1, and *Traj18* ^−/−^ (2-1) derived from Traj18_sgRNA2.

### Unbiased TCRα repertoire in *Traj18* mutant mice

**Figure 1:**
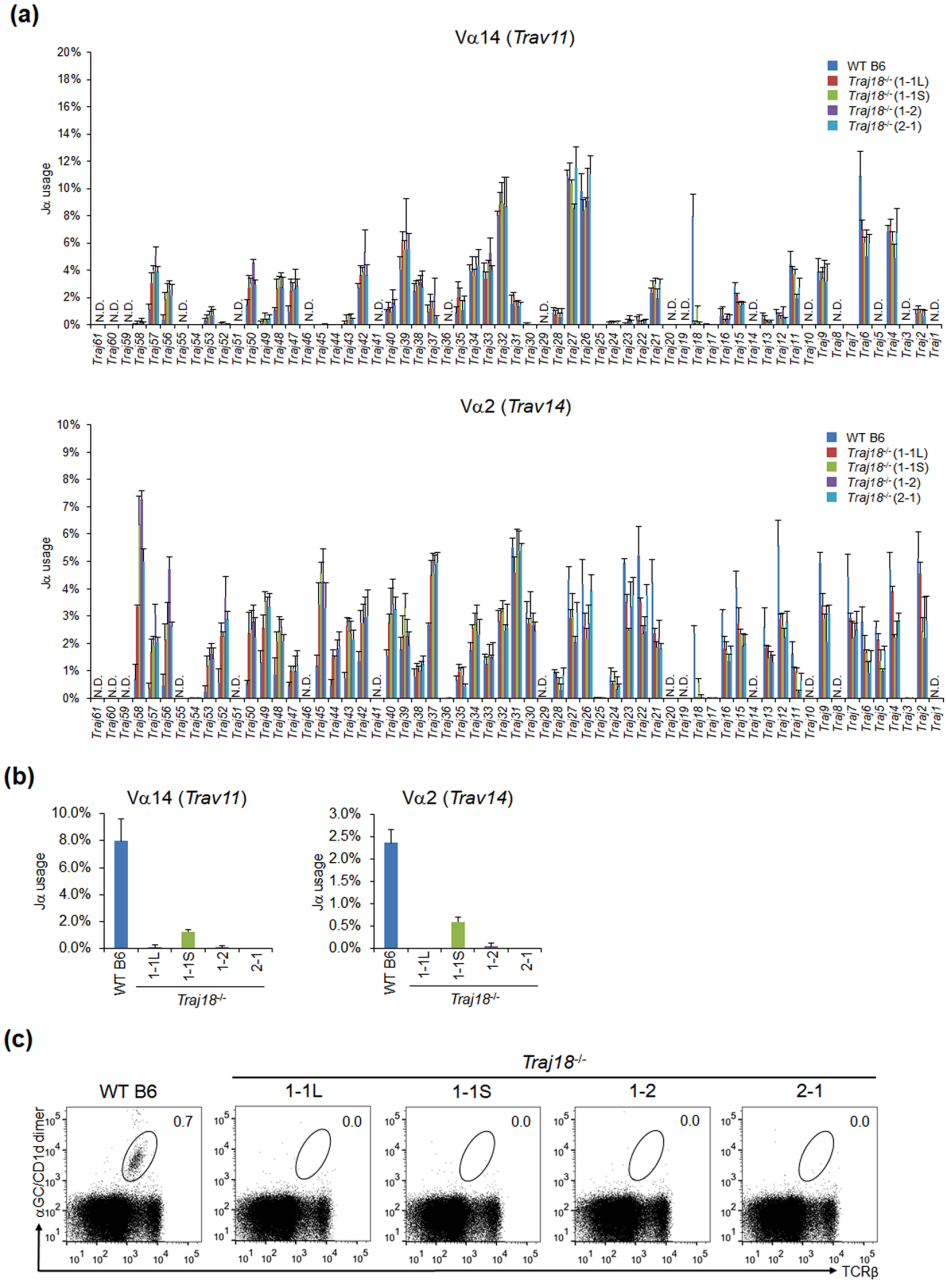
Generation of *Traj18* ^−/−^ mouse lines by CRISPR/Cas9 technology. **(a)** TCRα repertoire diversity analyzed by next generation sequencing. TCRβ^low^ CD4^+^ CD8^+^ CD69^−^ thymocytes from WT B6, *Traj18* ^−/−^ (1-1 L), *Traj18* ^−/−^ (1-1 S), *Traj18* ^−/−^ (1-2), and *Traj18* ^−/−^ (2-1) were sorted. *Trav11-Trac* or *Trav14-Trac* PCR products were prepared and subjected to next-generation sequencing analysis. The graphs show percentages of productive *Traj* gene segment rearrangements. Data represents mean ± SD of three biologically independent samples per group. **(b)** *Traj18* gene segment usage in *Trav11-Trac* or *Trav14-Trac* transcripts analyzed by next-generation sequencing. **(c)** Frequencies of iNKT cells (TCRβ^+^, α-GalCer/CD1d dimer^+^) in total thymocytes isolated from WT B6, *Traj18* ^−/−^ (1-1 L), *Traj18* ^−/−^ (1-1 S), *Traj18* ^−/−^ (1-2), and *Traj18* ^−/−^ (2-1) mice were analyzed by flow cytometry. Numbers represent the percentage of iNKT cells in the respective gates.

We compared the TCRα repertoire diversity in sorted pre-selection double-positive (DP) thymocytes (TCRβ^low^ CD4^+^ CD8^+^ CD69^−^) from *Traj18* ^−/−^ (1-1 L), *Traj18* ^−/−^ (1-1 S), *Traj18* ^−/−^ (1-2), *Traj18* ^−/−^ (2-1) and wild-type (WT) B6 mice. We performed PCR amplification of *Trav11* (encoded Vα14) that contains iNKT-TCRα, or *Trav14* (encoded Vα2), the most frequently used TCRα in αββT cells, by using a specific forward primer for each Vα encoding sequence and a reverse primer for the sequence encoding the TCRα constant region (Cα). The products were purified and subjected to next-generation sequencing analysis. All four *Traj18*-partial deletion mouse lines harbored similar *Traj* gene segments as WT B6 mice, except for *Traj18* (Fig. 1a). Selective deficiency in *Traj18* was confirmed in *Traj18* ^−/−^ (1-1 L), *Traj18* ^−/−^ (1-2), and *Traj18* ^−/−^ (2-1) lines, and we found a very low percentage of *Traj18* usage in *Traj18* ^−/−^ (1-1 S) mice (Fig. 1b). We further examined iNKT cells in the thymus by staining with α-GalCer-loaded CD1d-dimers, and confirmed the absence of iNKT cells in each of the four lines (Fig. 1c). These results demonstrated that the newly generated *Traj18*-partial deletion mouse lines lacked iNKT cells and harbored an undisturbed TCRα repertoire, fulfilling the criteria of iNKT cell-deficient mice.

### Novel *Traj18*^−/−^ mice are selectively deficient in iNKT cells

Since each of the four novel *Traj18* ^−/−^ mouse lines lacked thymic iNKT cells and contained a TCRα repertoire similar to WT mice, we next selected the *Traj18* ^−/−^ (1-1 L) line for further experiments.

**Figure 2:**
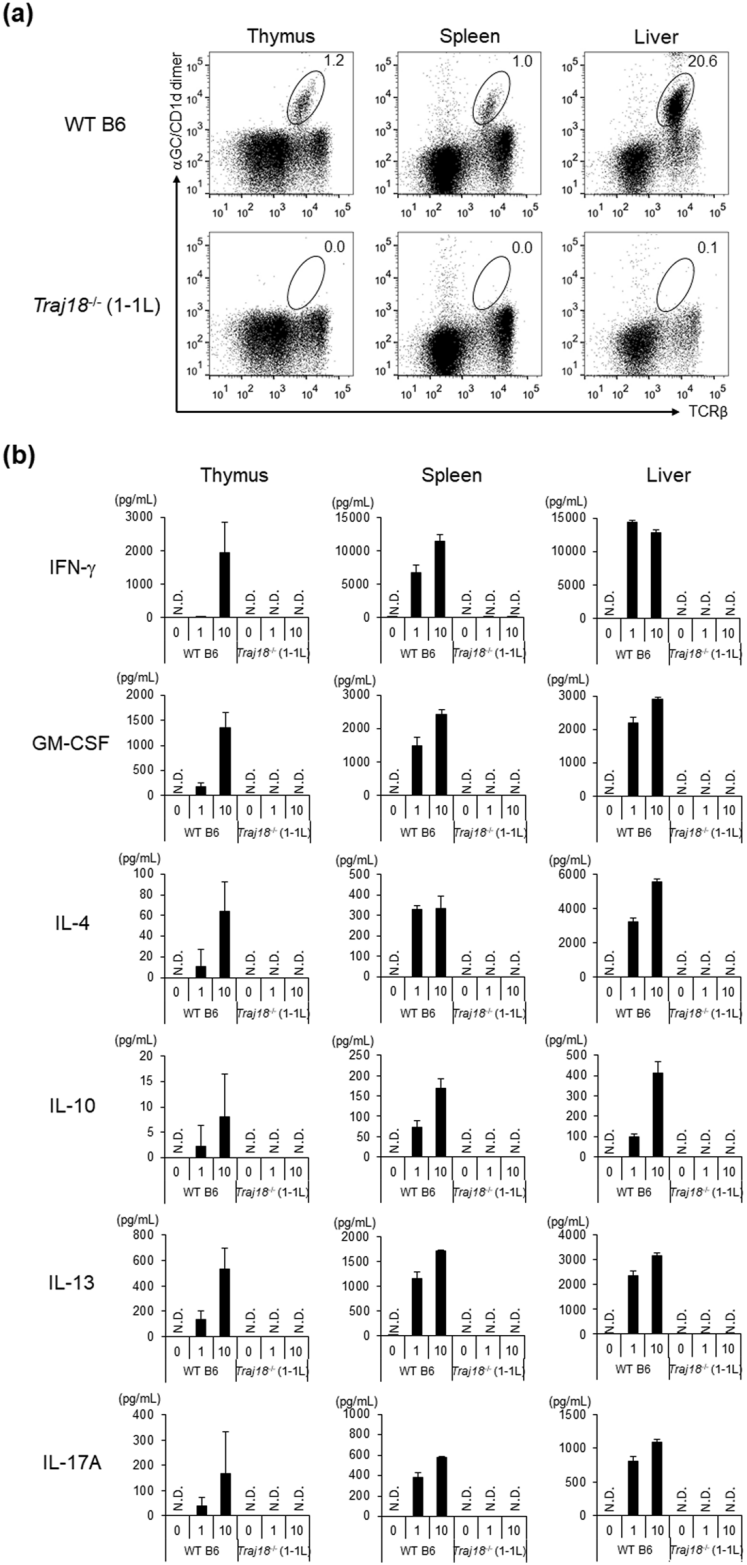
*Traj18* ^−/−^ (1-1 L) mice lack iNKT cells and fail to respond to α-GalCer stimulation. **(a)** Total thymocytes, splenocytes and liver MNCs isolated from WT B6 or *Traj18* ^−/−^ (1-1 L) mice were analyzed by flow cytometry. Numbers represent the frequencies of iNKT cells (TCRβ^+^, α-GalCer/CD1d dimer^+^) in the respective gates. **(b)** Total lymphocytes isolated from thymus (1 × 10^6^), spleen (1 × 10^6^), and liver (0.5 × 10^6^) of WT B6 or *Traj18* ^−/−^ (1-1 L) mice were stimulated with α-GalCer (0, 1 or 10 ng/mL), and the supernatants were collected 48 hrs post-stimulation. Cytokine levels of IFN-γ, GM-CSF, IL-4, IL-10, IL-13, and IL-17A were quantified by CBA. Data represents mean ± SD of each group (n = 3 per group). N.D., not detected.

Frequencies of iNKT cells in the spleen and liver from WT B6 and *Traj18* ^−/−^ (1-1 L) mice were analyzed by flow cytometry, which revealed iNKT cell-deficiency in *Traj18* ^−/−^ (1-1 L) mice (Fig. 2a). Analysis of developmental stages of thymocytes revealed no difference between *Traj18* ^−/−^ (1-1 L) and WT B6 mice (Supplemental Fig. 3a). We also analyzed the frequencies of T cells with specific functions such as type 2 NKT cells, regulatory T cells (Treg) and mucosal-associated invariant T (MAIT) cells in the thymus, resulting in no differences between *Traj18* ^−/−^ (1-1 L) and WT B6 mice (Supplemental Fig. 3b–d). Similarly, no differences were observed in the frequencies of other immune cell types in the spleen, including CD4/8 αβT cells, γδT cells, B cells, NK cells, conventional dendritic cells, plasmacytoid dendritic cells, macrophages, and granulocytes in the spleen of *Traj18* ^−/−^ (1-1 L) and WT B6 mice (Supplemental Fig. 4a–f), indicating that the development of these immune cells was not affected by the deletion of *Traj18* in the present mouse strain.

Levels of cytokines GM-CSF, IFN-γ, IL-4, IL-10, IL-13 and IL-17A, produced by total thymocytes, splenocytes and liver mononuclear cells (LMNCs) were measured after α-GalCer (0, 1, or 10 ng/mL) stimulation for 48 hrs, which revealed absence of cytokine production in *Traj18* ^−/−^ (1-1 L) mice (Fig. 2b). Similar loss of reactivity against α-GalCer was observed in other three lines, *Traj18* ^−/−^ (1-1 S), *Traj18* ^−/−^ (1-2) and *Traj18* ^−/−^ (2-1) (data not shown).

Because some functional studies on iNKT cells require animals on a genetic background distinct from the B6 strain, we also generated a *Traj18* ^−/−^ (1-1 L) BALB/c mouse line by backcrossing, and confirmed the absence of iNKT cells and cytokine production in response to α-GalCer stimulation (Supplemental Fig. 5a,b).

In summary, our data show that both B6 and BALB/c background *Traj18* ^−/−^ (1-1 L) strains are selectively iNKT cell-deficient.

### iNKT cell deficiency ameliorates high-fat diet-induced obesity

NKT cells are one of cell types that reside in adipose tissue. However, divergent findings for the metabolic role of iNKT cells have been reported in studies using the previously generated *Traj18* ^−/−^ mouse strain. Therefore, we re-investigated the contribution of iNKT cells to the development of obesity induced by a high-fat diet (HFD) using our novel *Traj18* ^−/−^ mouse strain on the B6 background. We first investigated the frequencies of type 2 NKT cells, Treg, MAIT cells, γδ T cells, M0/1/2 macrophages in *Traj18* ^−/−^ (1-1 L) in the steady state, because these immune cells are considered to be effector and regulatory cells in metabolic disorders. It was found that no differences were observed in frequencies of these immune cells between WT B6 and *Traj18* ^−/−^ (1-1 L) (Supplementary Fig. 6a–e).

**Figure 3:**
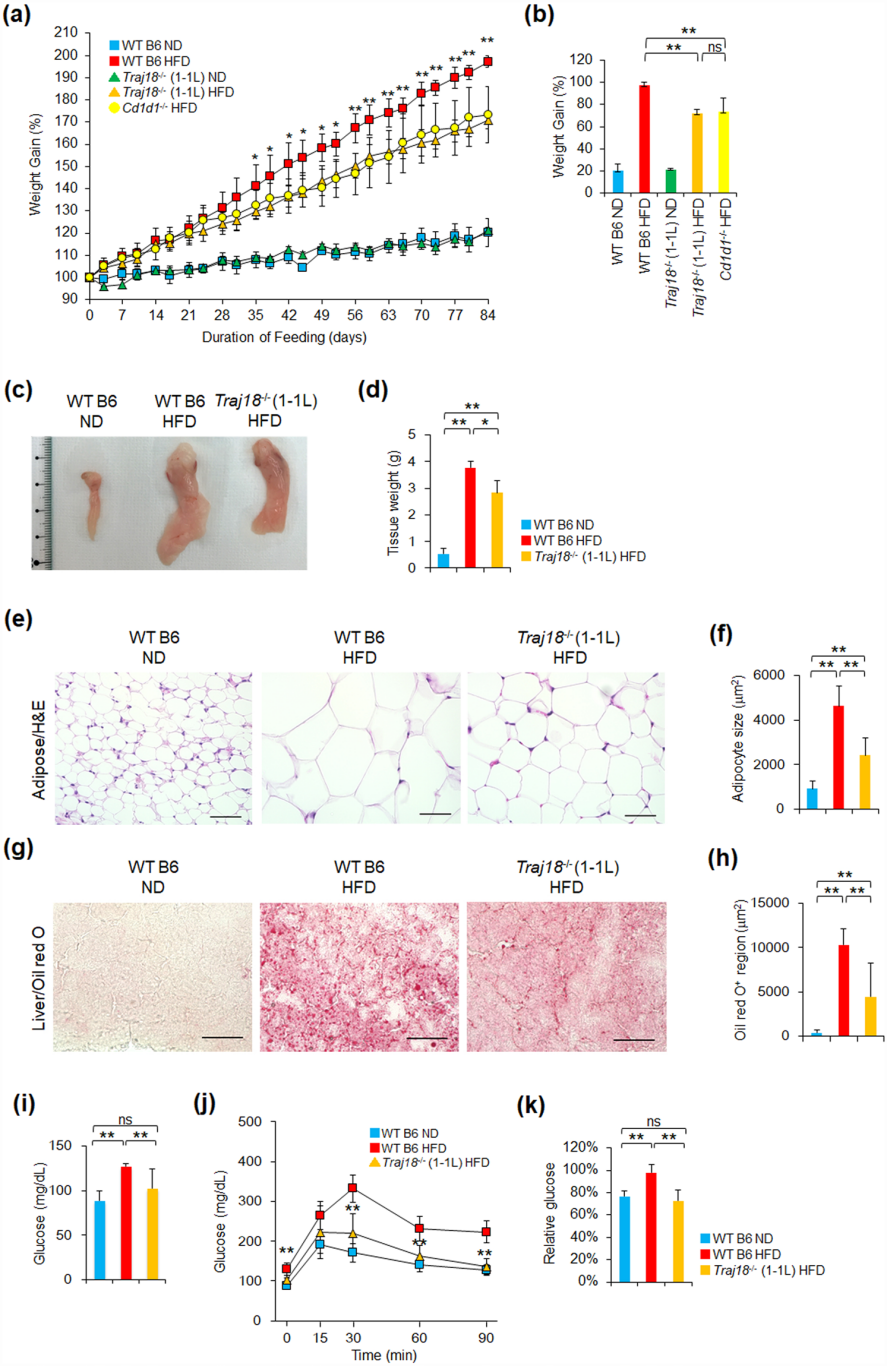
Impact of iNKT cell-deficiency on metabolic parameters. **(a)** Curve of relative body weight (BW_dn_/BW_d0_ × 100%) of WT B6 and *Traj18* ^−/−^ (1-1 L) mice on HFD or ND, as well as *Cd1d* ^−/−^ mice on HFD, for 84 days. Each point represents mean ± SD (n = 5 per group). **(b)** Overall relative weight gain ((BW_d84_ − BW_d0_)/BW_d0_ × 100%) after 84 days of HFD or ND. Data represents mean ± SD (n = 5 per group). **(c**,**d)** Photograph **(c)** and average weight **(d)** of visceral fat pad excised from WT B6 or *Traj18* ^−/−^ (1-1 L) mice after 91–98 days of HFD, as well as WT B6 on ND as control. Data represents mean ± SD of each group (n = 3 per group). **(e)** Histology of visceral adipose tissue from WT B6 or *Traj18* ^−/−^ (1-1 L) mice after 91-98 days of HFD, as well as WT B6 mice on ND, by H&E staining. Scale bars represent 50 μm. **(f)** Average size of adipocytes from each group was analyzed by image analysis software. Data represents mean ± SD of each group (n = 3 per group). **(g)** Histology of liver tissue from WT B6 or *Traj18* ^−/−^ (1-1 L) mice after 91-98 days of HFD, as well as WT B6 mice on ND, by Oil-Red-O staining. Scale bars represent 50 μm. **(h)** The average area of Oil-Red-O-stained lipid droplets in liver from each group was analyzed by image analysis software. **(i)** Fasting glucose levels of WT B6 or *Traj18* ^−/−^ (1-1 L) mice on HFD, as well as WT B6 mice on ND, for 84 days. Data represents mean ± SD of each group (n = 3 per group). **(j)** GTT results of WT B6 or *Traj18* ^−/−^ (1-1 L) mice on HFD, as well as WT B6 mice on ND, for 84 days. One g/kg of glucose was i.p. injected into overnight-fasted mice, and glucose levels were measured at 0, 15, 30, 60, and 90 mins. Data represents mean ± SD of each group (n = 3 per group). **(k)** ITT results of WT B6 or *Traj18* ^−/−^ (1-1 L) mice on HFD, as well as WT B6 mice on ND, for 91 days. Insulin (0.75 U/kg) was i.p. injected into fasted (5 hrs) mice, and glucose levels were measured at 0 and 15 mins. The graph shows relative glucose (Glucose_Tn_/Glucose_T0_ × 100%). Data represents mean ± SD of each group (n = 3 per group). *p < 0.05; **p < 0.01; ns, no significantly difference. The results are representative of three independent experiments.

WT B6, *Traj18* ^−/−^ (1-1 L), and *Cd1d* ^*−/−*^ male mice were fed with a HFD or a normal chow diet (ND) starting from 8 weeks of age. For WT B6 and *Traj18* ^−/−^ (1-1 L) mice receiving ND, similar weight curves were observed during the 84-day feeding period. All mouse strains receiving HFD showed more substantial weight gain than those receiving ND throughout the feeding period. Among the experimental groups on HFD, both *Traj18* ^−/−^ (1-1 L) and *Cd1d* ^*−/−*^ mice gained less weight than WT B6 mice, whereas there was no significant difference in the weight gain between *Traj18* ^−/−^ (1-1 L) and *Cd1d* ^*−/−*^ mice (Fig. 3a,b).

The size and mass of the visceral fat-pads from WT B6 mice were substantially larger than those from *Traj18* ^−/−^ (1-1 L) mice (Fig. 3c,d). Similarly, the size of adipocytes from *Traj18* ^−/−^ (1-1 L) mice on HFD was smaller than that of WT B6 mice (Fig. 3e,f). Furthermore, the livers of *Traj18* ^−/−^ (1-1 L) mice on HFD contained significantly less fat deposits compared to WT B6 mice on HFD, as revealed by distinctly smaller Oil-Red-O-stained areas (Fig. 3g,h).

### iNKT cell-deficiency ameliorates glucose intolerance and insulin resistance

We next performed glucose tolerance tests (GTT) and insulin tolerance tests (ITT) to investigate whether iNKT cell-deficiency influences glucose and insulin tolerance in mice. We detected increased fasting blood glucose levels in WT B6 mice compared with *Traj18* ^−/−^ (1-1 L) mice on HFD (Fig. 3i). Following injection of glucose, WT B6 mice on HFD showed rapidly increasing blood glucose levels, which remained substantially elevated throughout the 90-min measurement period, indicating impaired glucose tolerance. Interestingly, improved glucose tolerance was observed in obese *Traj18* ^−/−^ (1-1 L) mice, which presented with similar changes in blood glucose levels as WT B6 mice on ND (Fig. 3j). During ITT measurement, unchanged blood glucose levels were observed in WT B6 mice at 15 mins after insulin challenge, whereas glucose levels in similarly treated obese *Traj18* ^−/−^ (1-1 L) mice were reduced to levels similar as WT B6 on ND (Fig. 3k). Thus, the ITT results revealed insulin-resistance in obese WT B6 mice, but not in obese *Traj18* ^−/−^ (1-1 L) mice.

Taken together, the novel *Traj18* ^−/−^ mouse strain exhibits ameliorated metabolic phenotypes, which is consistent with a pathogenic role of iNKT cells in the development of obesity and insulin-resistance.

## Discussion

T cells in the widely used *Traj18* ^−/−^ mouse strain that was generated by traditional embryonic stem cell/gene targeting technology contain a disturbed TCRα repertoire due to altered usage of *Traj* gene segments upstream of *Traj18*, where the *PGK-Neo* ^r^ cassette employed to replace the *Traj18* allele might have caused inadvertent alterations in TCR gene transcription and rearrangement^7^. This impaired TCRα repertoire diversity might have resulted in the loss of some unique T cell subsets, raising concerns about experimental results obtained with this mouse strain. To avoid the unintended consequences caused by the *PGK-Neo* ^r^ system on other *Traj* gene segments, we and other groups^12–14^ tried to generate *Traj18* null mice with an undisturbed TCRα repertoire. Two groups^12,13^ described *Traj18* deletion mice created on the C57BL/6 background in which *Neo* ^*r*^ was deleted along with the *Traj18* gene segment by traditional homologous recombination in C57BL/6 ES cells with Cre/loxP and/or FLP/FRT method. Zhang *et al*.^14^ created using TALEN methodology to only disrupt the expression of Jα18, leaving the remaining Jα repertoire unperturbed. The mice have 10 bp deletion in *Traj18* exon resulting in lack of iNKT cells even in the existence of *Trav11*-*Traj18* transcript, highlighted the importance of the CDR3 sequence of iNKT-TCRα in the recognition of α-GalCer/CD1d. Here we employed another genome editing CRISPR/Cas9 technology to generate four strains of *Traj18* null mice with C57BL/6 background. We also created *Traj18* null mice with BALB/c background from *Traj18* ^−/−^ (1-1 L) by backcrossing. Importantly, these mice contained profoundly reduced *Trav11-Traj18* TCRα mRNA and lacked α-GalCer/CD1d-restricted iNKT cells. Although the minor population of α-GalCer/CD1d-restricted cells with a Vα10-Jα50 (*Trav13-Traj50*) rearrangement^16^ would not be affected by deletion of *Traj18*, we did not find definitive evidence for the presence of these cells by PCR (data not shown) or FACS (Fig. 1c), similar to results reported previously^12^. We confirmed the presence of an unbiased TCRα repertoire by analyzing the frequency of Jα usage for *Trav11* and *Trav14*.

NKT cells, indicating both type 1 iNKT cells and type 2 NKT cells, are one of the adipose tissue-resident immune cell subsets that have been implicated in the development of obesity and related metabolic conditions^5^. However, divergent data have been obtained regarding the metabolic phenotype of NKT cell-deficient mice. Lynch *et al*. found evidence for a protective role of NKT cells against obesity, reporting worsened metabolic parameters in both *Traj18* ^−/−^ and *Cd1d* ^*−/−*^ mice^9^. In contrast, Wu *et al*.^10^ suggested a pathogenic role of NKT cells by showing ameliorated hepatic steatosis, glucose tolerance and insulin sensitivity, as well as reduced tissue inflammation in *Traj18* ^−/−^ and *Cd1d* ^*−/−*^ mice. Pathological roles of NKT cells in obesity were also reported by Satoh *et al*.^11^ in *Cd1d* ^*−/−*^ mice, but these investigators found no difference in the metabolic parameters between *Traj18* ^−/−^ and WT B6 mice on HFD feeding, arguing for a pathogenic role of type 2 rather than type 1 iNKT cells. There might be many reasons for these divergent results, such as differences in the type of HFD, the age and gender of the mice, and differences in the gut flora or environmental microbial distribution among the animals employed by the different research groups. It is interesting to note that iNKT cells can influence microbial colonization in the gut^17^ and, conversely, that the normal microbiota can impact iNKT cell numbers and functions^18–20^. Another confounding factor in interpreting these findings is the impaired TCR diversity of the *Traj18* ^−/−^ mouse line used in the previous studies. Interestingly, one of the affected T cell populations, MAIT cells that uses the invariant Vα19 Jα33 chain (encoded by *Trav1-Traj33*) in mice^21^, were recently reported to have altered distribution and cytokine production in obese patients, and were found to be positively associated with insulin resistance^22,23^. Considering the potential role of MAIT cells and other T cell subsets in obesity, results obtained with the *Traj18* ^−/−^ mouse model employed in the previous studies need to be interpreted with caution. In the present study, we have re-investigated the contribution of iNKT cells to obesity using our new *Traj18* ^−/−^ mouse strain, *Traj18* ^−/−^ (1-1 L). These *Traj18* ^−/−^ (1-1 L) mice have a selective deletion in the *Traj18* locus, causing a selective loss in iNKT cells, leaving the remaining TCRα repertoire intact and less affecting in the development and generation of other immune cells including MAIT cells and Treg at least in the steady state. Our findings with *Traj18* ^−/−^ (1-1 L) mice revealed ameliorated metabolic phenotypes, including reduced weight gain as compared with WT B6 mice of HFD. It is notable that increased or similar levels of weight gain in *Traj18* ^−/−^ mice as compared with WT B6 mice on HFD were observed in almost all of the previous metabolic studies^9–11^. Although differences in animal diets and gut microbiota cannot be excluded as confounding factors, the reduced weight gain observed for *Traj18* ^−/−^ (1-1 L) mice employed in our studies might represent a physiologically distinct phenotype as that for the findings with *Traj18* ^−/−^ mice employed in the previous studies. The content of iNKT1 and iNKT2 cell subtypes is known to be different between B6 and BALB/c background^24^. It is also important to compare cell numbers and functional status of Treg, MAIT cells and also M1/M2 macrophages in adipose tissues between the new strain of *Traj18*-deficient mice with both background and their WT counterparts fed after HFD.

In addition to the new findings with *Traj18* ^−/−^ (1-1 L) mice, we observed similar weight gain curves for *Traj18* ^−/−^ (1-1 L) and *Cd1d* ^*−/−*^ mice on HFD, which would be consistent with a limited role of type 2 NKT cells in the development of obesity. However, an over-representation of MAIT cells was recently observed in *Cd1d* ^*−/−*^ mice^25^. We found similar number of MAIT cells in the thymus and steady state subcutaneous adipose tissue by FACS. Another new *Traj18* ^−/−^ mouse line generated by Dashtsoodol *et al*. also contained similar *Trav1-Traj33* expression levels as WT B6 mice^13^, indicating normal MAIT cell development and cell number. As such, differences in MAIT cell numbers between *Traj18* ^−/−^ (1-1 L) and *Cd1d* ^*−/−*^ mice might influence conclusions about the role of type 2 NKT cell functions in metabolic disease parameters. Thus, further studies comparing the development and function of MAIT cells in the different animal models of NKT cell-deficiency are needed.

Taken together, we have successfully generated new *Traj18* ^−/−^ mouse strains carrying an undisturbed TCRα repertoire by CRISPR/Cas9 technology. Using this new mouse strain, we re-assessed the contribution of iNKT cells to obesity and its associated metabolic abnormalities, and found that *Traj18*–deficient mice showed reduced weight gain and ameliorated metabolic parameters. Our present data suggest a pathogenic role of iNKT cells in the development of obesity and insulin-resistance.

## Methods

### Mice

B6 and BALB/c mice were purchased from SLC Japan, Inc. For obesity experiments, mice received ND (13.7 fat kcal%, CA-1, CLEA) or HFD (58 fat kcal%, D12331, Research Diets) from 8 weeks of age, for a time period of approximately 12 weeks. All mice were kept under specific pathogen-free conditions and were used at 8-24 weeks of age. All animal experiments were in accordance with protocols approved by the Animal Care and Use Committee, The University of Tokyo. *Cd1d* ^−/−^ mice^26^ were kindly provided by Dr. Luc Van Kaer (Vanderbilt University).

### Plasmids and mRNA preparation

To generate the pCAG-EGxnFP reporter plasmid, the N-terminal and C-terminal EGFP coding regions were PCR-amplified and placed under control of the ubiquitous CAG promoter, as inserted into the Xho I and Not I restriction enzyme sites. The ~500 bp genomic fragments containing sgRNA target sequence were PCR-amplified and placed between the EGFP fragments. sgRNAs were designed by CRISPRdirect (https://crispr.dbcls.jp/) and selected Traj18_sgRNA1 and Traj18_sgRNA2 based on the specificity and GC content (>50%). Plasmids expressing sgRNA were prepared by ligating oligos directionally into BbsI restriction enzyme sites upstream of the long gRNA sequence^27^. The plasmid expressing hCas9, pCAG-T3-hCas9-pA, was licensed and purchased from Addgene (http://www.addgene.org/48625/). As for RNA preparation, sgRNA were placed downstream of the T3 promoter in pUC19 plasmids. The resulting plasmids were subjected to RNA synthesis with mMESSAGE mMACHINE T7 kit (Ambion) according to the manufacturer’s protocol. The hCas9 mRNA and sgRNAs were purified by RNeasy kit (QIAGEN).

### Transfection

Five hundred ng of pCAG-EGxnFP-target was mixed with 500 ng of pCAG-T3-hCas9-pA and with 500 ng of pU6-sgRNA including sgRNA sequences (Traj18_sgRNA) or with pU6-sgRNA vector itself as a control, and then introduced into 4 × 10^5^ HEK293T cells/well in six-well plates by FuGene HD (Promega) according to the manufacturer’s protocol. EGFP fluorescence was observed under a fluorescence microscope (Zeiss) and using flow cytometry with a FACS Calibur (BD Biosciences) at 48 hrs after transfection.

### Generation of *Traj18*^−/−^ mice

The synthesized sgRNAs (10–50 ng/μL) and hCas9 mRNA (10–50 ng/μL) were injected into the pronucleus of fertilized eggs from B6 mice. The *Traj18* locus in all of the pups derived from the injected zygotes were genotyped and sequenced using forward primer 5′-ACTGTCTACCGTAGGCTGCTG-3′ and reverse primer 5′-TCATGGTAATGTTTCCCTGGA-3′. Pups that harbored the defective *Traj18* gene in their genome were chosen for further breeding to generate the *Traj18*-deficient mouse line. After crossing with B6 mice and generating homozygous mice, *Traj18* ^−/−^ (1-1 L), *Traj18* ^−/−^ (1-1 S), *Traj18* ^−/−^ (1-2), and *Traj18* ^−/−^ (2-1) lines were established. *Traj18* ^−/−^ (1-1 L) B6 mice were backcrossed for over 8 generations with WT BALB/c mice and then intercrossed to generate *Traj18* ^−/−^ mice with a BALB/c background. We would like to declare the availability of the generated mice under material transfer agreement.

### TCR sequencing

TCRβ^low^ CD4^+^ CD8^+^ CD69^−^ thymocytes were sorted on FACSAria III (BD Biosciences). Cells were lysed and RNA was extracted with a Qiagen RNeasy Micro kit. cDNA was prepared from RNA with a Superscript III reverse transcriptase kit (Life Technologies). PCR was performed for amplification of the *Trav11-Trac* or *Trav14-Trac* gene segments with specific primers, each with an Illumina adaptor sequence on the 5′ end (*Trav11*, GTCCTCAGTCCCTGGTTGTC; *Trav14*, TGCAGTTATGAGGACAGCACTT; *Trac*, AGGGTGCTGTCCTGAGACCGA) by using KOD plus Neo (Toyobo). PCR products were purified by QIAquick PCR purification kit (QIAGEN) and analyzed by Agilent 2200 TapeStation (Agilent Technologies), followed by sequencing on a MiSeq system with MiSeq Reagent Kit v2-500 (Illumina). The sequence reads were mapped to the mouse genome using MiXCR^28^. Mouse TCR Jα regions were analyzed using the IMGT (international ImMunoGeneTics information system) database. The raw sequence data were registered at the DNA Data Bank of Japan (DDBJ) under the accession number DRA005788.

### Flow cytometry

Antibodies (BD Biosciences, eBioscience or BioLegend) used for flow cytometry detection were: FITC anti-TCRβ (H57-597), FITC anti-CD38 (90), APC anti-CD8α (53-6.7), APC anti-F4/80 (BM8), APC anti-Egr2 (erongr2), APC-Cy7 anti-CD19 (6D5), APC-Cy7 anti-TCRβ (H57-597), APC-Cy7 anti-CD4 (RM4-5), Pacific Blue anti-CD8αα (53-6.7), PE-Cy7 anti-NK1.1 (PK136), PE-Cy7 anti-CD11b (M1/70), PE anti-TCRγδ (GL3), PE anti-CD11c (N418), PE anti-CD69 (H1.2F3), PE anti-NK1.1 (PK136), PE anti-FoxP3 (NRRF-30), PerCP-Cy5.5 anti-B220 (RA3-6B2), PerCP-Cy5.5 anti-CD4 (RM4-5), V500 anti-CD4 (RM4-5), Pacific Blue anti-Gr-1 (RB6-8C5), BV421 anti-F4/80 (BM8). α-GalCer-loaded CD1d (α-GalCer/CD1d) dimer for iNKT cell enrichment and detection were prepared by the method described previously^29^. 5-OP-RU or 6-FP loaded MR1 tetramer for MAIT cell detection was kindly provided by NIH Tetramer Core Facility. Cells were analyzed and sorted by FACSAria III (BD Biosciences). Data were analyzed by FlowJo software (Tree Star). The flow diagrams in the figures are representative of at least three experiments.

### Cytokine measurement

Total lymphocytes (1 × 10^6^ for thymus and spleen, and 0.5 × 10^6^ for liver) were isolated from mice and were stimulated with 0, 1 or 10 ng/mL of α-GalCer. Supernatants were collected after 48hrs incubation. Concentrations of GM-CSF, IFN-γ, IL-4, IL-10, IL-13, and IL-17A were measured by Cytometric Bead Array (CBA; BD Biosciences), according to the manufacturer’s instructions. Data were analyzed by FCAP software (BD Biosciences).

### Metabolic studies

All mice were weighted 2 times per week, and food intake was monitored. After 12 weeks of HFD, GTT and ITT were carried out. For GTT, overnight fasted mice received glucose at 1 g/kg bodyweight (BW) intraperitoneally (i.p.), and glucose levels were measured at 0, 15, 30, 60, and 90 min time points (Terumo). For ITT, mice were fasted for 5 hrs before receiving insulin (Wako) at 0.75 U/kg BW i.p., and glucose levels were measured at 0 and 15 min time points. Adipose tissue was collected, weighed and fixed in formalin prior to paraffin mounting, sectioning and staining with hematoxylin and eosin (H&E). Livers were collected after perfusion with PBS. Parts of liver tissue were frozen in Tissue-Tek OCT compound (Sakura Finetek) in liquid nitrogen and sections were stained with Oil-Red-O (Sigma) for measurement of fat deposition. The degree of fat deposition was measured by Oil-Red-O staining intensity around three portal-tract areas per slide.

### Statistical Analysis

Data were presented as mean ± S.D. from three independent experiments. The statistical significance of differences was determined by the Student’s t-test or two-way ANOVA. Differences with a *p*-value of < 0.05 were considered significant (*p < 0.05; **p < 0.01).

## Electronic supplementary material

FigS1 to S6, TableS1
