# Supplementary material for: A Novel Mouse Model of iNKT Cell-deficiency Generated by CRISPR/Cas9 Reveals a Pathogenic Role of iNKT Cells in Metabolic Disease
Source: Sci Rep. 2017 Oct 6;7:12765. doi: 10.1038/s41598-017-12475-4 (PMC5630609; doi:10.1038/s41598-017-12475-4)
Supplement: Supplementary file 1 — FigS1 to S6, TableS1 [file 41598_2017_12475_MOESM1_ESM.pdf]

Supplementary Information

A Novel Mouse Model of iNKT Cell-deficiency Generated by CRISPR/Cas9 Reveals a Pathogenic Role of iNKT Cells in Metabolic Disease

Yue Ren[1,2], Etsuko Sekine-Kondo[1], Risa Shibata[1,3], Megumi Kato-Itoh[4], Ayumi Umino[4], Ayaka Yanagida[4,*], Masashi Satoh[5], Komaki Inoue[6], Tomoyuki Yamaguchi[4], Keiichi Mochida[6], Susumu Nakae[3], Luc Van Kaer[7], Kazuya Iwabuchi[5], Hiromitsu Nakauchi[4,8], Hiroshi Watarai[1,**]

[1] Division of Stem Cell Cellomics, Center for Stem Cell Biology and Regenerative Medicine, Institute of Medical Science, University of Tokyo, Minato-ku, Tokyo, Japan.

[2] The Neurological Institute of Jiangxi Province, Department of Neurology, Jiangxi Provincial People's Hospital, Nanchang, Jiangxi, China.

[3] Laboratory of Systems Biology, Center for Experimental Medicine and Systems Biology, Institute of Medical Science, University of Tokyo, Tokyo, Japan.

[4] Division of Stem Cell Therapy, Center for Stem Cell Biology and Regenerative Medicine, Institute of Medical Science, University of Tokyo, Tokyo, Japan.

[5] Department of Immunology, Kitasato University School of Medicine, Sagamihara, Japan.

[6] Cellulose Production Research Team, RIKEN Center for Sustainable Resource Science, Yokohama, Japan.

[7] Department of Pathology, Microbiology and Immunology, Vanderbilt University School of Medicine, Nashville, Tennessee, USA

[8] Institute for Stem Cell Biology and Regenerative Medicine, Department of Genetics, Stanford University School of Medicine, Stanford, California, USA

*) current affiliation: Wellcome Trust-Medical Research Council Cambridge Stem Cell Institute, University of Cambridge, Cambridge CB2 1QR, UK

**) Correspondence to hwatarai@ims.u-tokyo.ac.jp

Supplemental Figure Legend

Fig. S1 CRISPR/Cas9-mediated *Traj18* deletions *in vitro*. (a) Target sequence of each sgRNA in the *Traj18* locus (bold: *Traj18* exon, underline: sgRNA recognition site, box: PAM sequence). **(b)** Genomic fragment containing the sgRNA target sequence placed between EGFP fragments of the pCAG-EGxnFP plasmid (bold: *Traj18* exon). **(c)** Plasmids used in this study. pU6-sgRNA plasmid contains BbsI sites that enables directional cloning of sgRNA oligos. pCAG-EGxnFP contains cloning sites (XhoI and NotI). **(d,e)** The efficiency of double-strand break-mediated homology-dependent repair was validated by observing EGFP fluorescence 48 hrs after the transfection by **(d)** fluorescence microscopy (bars: 50 μm) and **(e)** flow cytometry.

Fig. S2 CRISPR/Cas9-mediated *Traj18* deletions in mice. (a) Synthesized sgRNAs and hCas9 mRNA by T3 RNA polymerase. **(b,c)** Sequencing results of targeted locus deletion in all founder mice generated from injection of hCas9 mRNA with Traj18_sgRNA1 **(b)** or Traj18_sgRNA2 **(c)**. bold: *Traj18* exon, underline: sgRNA recognition site.

Fig. S3 Normal development of T cells other than iNKT cells in the thymus of *Traj18*$^{-/-}$ (1-1L) mice. Total thymocytes isolated from WT B6 or *Traj18*$^{-/-}$ (1-1L) mice were analyzed by flow cytometry in α-GalCer/CD1d dimer^{-} gates. **(a)** Numbers represent the frequencies of CD4^{+} SP (CD4^{+}, CD8^{-}), CD8^{+} SP (CD4^{-}, CD8^{+}), DP cells (CD4^{+}, CD8^{+}) and DN cells (CD4^{-}, CD8^{-}) were analyzed. **(b)** Numbers represent the frequencies of TCRβ^{+} NK1.1^{+} type 2 NKT cells. CD4^{+} SP (CD4^{+}, CD8^{-}), CD8^{+} SP (CD4^{-}, CD8^{+}), and DN cells (CD4^{-}, CD8^{-}) were further analyzed. **(c, d)** Numbers represent the frequencies of **(c)** regulatory T cells (CD4^{+}, FoxP3^{+}), **(d)** MAIT cells showing reactivity to 5-OP-RU/MR1 tetramer or 6-FP/MR1 tetramer.

Fig. S4 Normal development of immune cells other than iNKT cells in the spleen of *Traj18*$^{-/-}$ (1-1L) mice. Total splenocytes isolated from WT B6 or *Traj18*$^{-/-}$ (1-1L) mice were analyzed by flow cytometry. Numbers represent the frequencies of **(a)** CD4 T cells ($CD4^{+}$, $CD8^{-}$) and CD8 T cells ($CD4^{-}$, $CD8^{+}$) in $B220^{-}$ α-GalCer/CD1d $dimer^{-}$ gates. **(b-f)** Numbers represent the frequencies of other immune cells in α-GalCer/CD1d $dimer^{-}$ gates. **(b)** B cells ($B220^{+}$, $TCR\beta^{-}$), **(c)** natural killer cells ($NK1.1^{+}$, $TCR\beta^{-}$), **(d)** γδ T cells ($TCR\gamma\delta^{+}$, $TCR\beta^{-}$), **(e)** conventional dendritic cells ($CD11c^{hi}$, $B220^{-}$) and plasmacytoid dendritic cells ($CD11c^{int}$, $B220^{+}$), as well as **(f)** granulocytes ($CD11b^{hi}$, $Gr\text{-}1^{+}$) and macrophages ($CD11b^{int}$, $Gr\text{-}1^{-}$) in the respective gates.

Fig. S5 *Traj18*$^{-/-}$ (1-1L) BALB/c mice lack iNKT cells and fail to react to α-GalCer stimulation. **(a)** Total thymocytes, splenocytes and liver MNCs isolated from WT BALB/c or *Traj18*$^{-/-}$ (1-1L) mice were analyzed by flow cytometry. Numbers represent the frequencies of iNKT cells ($TCR\beta^{+}$, α-GalCer/CD1d $dimer^{+}$) in the respective gates. **(b)** Total lymphocytes isolated from thymus (1×10^{6}), spleen (1×10^{6}), and liver (0.5×10^{6}) of WT BALB/c or *Traj18*$^{-/-}$ (1-1L) mice were stimulated with α-GalCer (0, 1 or 10 ng/mL), and the supernatants were collected 48 hrs post stimulation. Cytokine levels of IFN-γ, GM-CSF, IL-4, IL-10, IL-13, and IL-17A were quantified by CBA. Data represents mean±SD of each group (n=3 per group). N.D. , not detected.

Fig. S6 Normal existence of T cells and macrophages other than iNKT cells in the adipose tissue of *Traj18*$^{-/-}$ (1-1L) mice in steady state. Total subcutaneous adipose tissue isolated from WT B6 or *Traj18*$^{-/-}$ (1-1L) mice were analyzed by flow cytometry. **(a)** Numbers represent the frequencies of iNKT cells ($TCR\beta^{+}$ α-GalCer/CD1d $dimer^{+}$). **(b)** Numbers represent the frequencies of $TCR\beta^{+}$ $NK1.1^{+}$ type 2 NKT cells in α-GalCer/CD1d $dimer^{-}$ population. $CD4^{+}$ SP ($CD4^{+}$, $CD8^{-}$),

CD8$^+$ SP (CD4$^-$, CD8$^+$), and DN cells (CD4$^-$, CD8$^-$) were further analyzed. **(c-e)** Numbers represent the frequencies of **(c)** regulatory T cells (CD4$^+$, FoxP3$^+$), **(d)** MAIT cells showing reactivity to 5-OP-RU/MR1 tetramer or 6-FP/MR1 tetramer. **(e)** Numbers represent the frequencies of CD11b$^+$ F4/80$^+$ macrophages in α-GalCer/CD1d dimer$^-$ population. M0 (CD38$^-$, Egr2$^-$), M1 (CD38$^+$, Egr2$^{+/-}$), M2 (CD38$^-$, Egr2$^+$) macrophages were further analyzed.

Supplemental Table

Table S1. Summary of in vitro fertilization

	Traj18_sgRNA1	Traj18_sgRNA2
No. of injected embryo	110	110
No. of live embryo	106 (96.3%)	106 (96.3%)
No. of 2 cell stage embryo	98 (92.4%)	89 (83.9%)
No. of transplantation	106	106
No. of implantation	51 (48.1%)	49 (46.2%)
No. of offspring	14 (13.2%)	18 (17.0%)
No. of weaning	11	17
No. of genome edited	9	11

(a)

GGAGCTGGGACTCAGCTGATTGTCATACCTGGTGAGTAGGTTTCTTTCTACTCCAGGGAAACATTA
Traj18_sgRNA1

GGAGCTGGGACTCAGCTGATTGTCATACCTGGTGAGTAGGTTTCTTTCTACTCCAGGGAAACATTA
Traj18_sgRNA2

(b)

ACTGTCTACCGTAGGCTGCTGGGTGCTTGGGGGAAAGACTTAGGTAAGCTCAGAGCGGACAGAAGA
TCGTTTTCGGCCCTACACTCCCAAGCCAGGGAGAGAGGCTGGGGAATGGTAGGAGCAGATTTGTGT
AAAGGGGGCTGGCACTGTG**TAGATAGAGGTTCAGCCTTAGGGAGGCTGCATTTTGGAGCTGGGACT**
CAGCTGATTGTCATACCTGGTGAGTAGGTTTCTTTCTACTCCAGGGAAACATTACCATGA

(c)

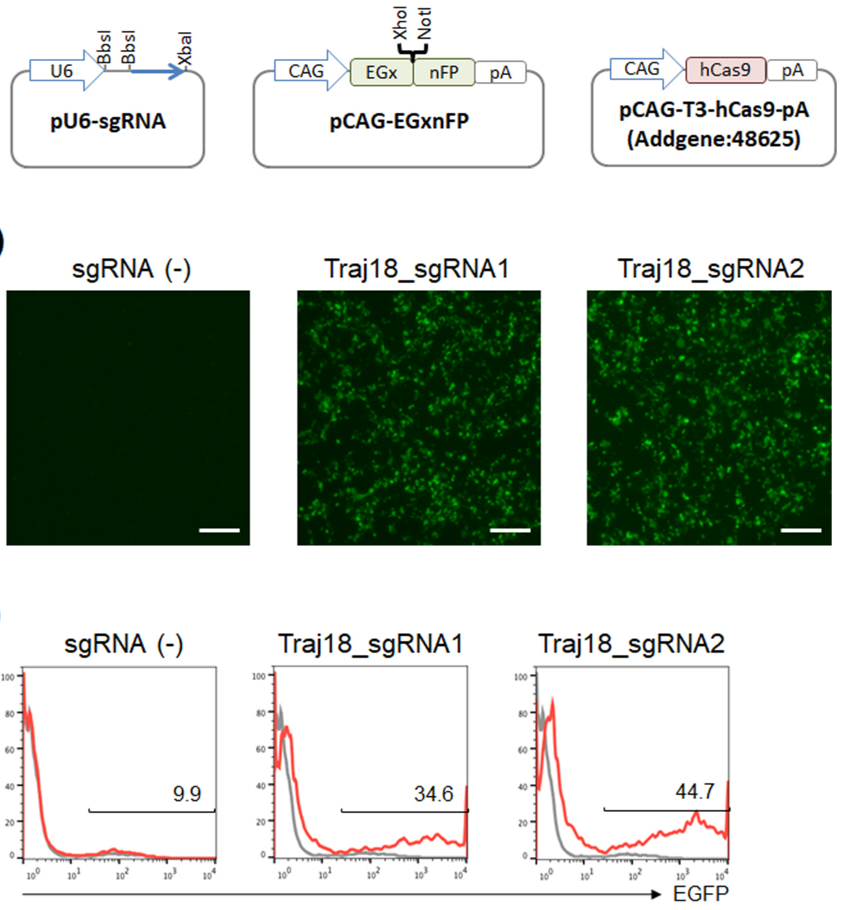

Fig.S2

(a)

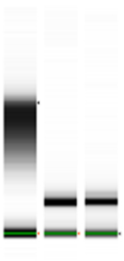

1: hCas9 mRNA
2: Traj18_sgRNA1
3: Traj18_sgRNA2

(b)

```
WT              GGAGCTGGGACTCAGCTGATTGTCATACCTGGTGAGTAGGTTTCTTTCTACTCCAGGGAAACATTACC
1-1L(♂,-/-)     GGAGCTGGGACTCAGCTGATT---------------------------------------(116bp)
1-1S(♂,-/-)     GGAGCTGGGACTCAGCTGATT----------GTGAGTAGGTTTCTTTCTACTCCAGGGAAACATTACC
1-2(♂,-/-)      GGAGCTGGGACTCAGCTGA----------------GTAGGTTTCTTTCTACTCCAGGGAAACATTACC
1-3(♂,-/-)      GGAGCTGGGACTCAGCTGATTGTCC--CCTGGTGAGTAGGTTTTTTTCTACTCCAGGGAAACATTACC
1-4(♂,-/-)      GGAGCTGGGACTCAGCTGATTGTCA--CCTGGTGAGTAGGTTTCTTTCTACTCCAGGGAAACATTACC
1-5(♂,+/+)      GGAGCTGGGACTCAGCTGATTGTCATACCTGGTGAGTAGGTTTCTTTCTACTCCAGGGAAACATTACC
1-6(♀,-/-)      GGAGCTGGGACTCAGCTGACTGGAG----TGGTGAGTAGGTTTCTTTCTACTCCAGGGAAACATTACC
1-7(♀,-/-)      GGAGCTGGGACTCACCTG-------------GTGAGTAGGTTTCTTTCTACTCCAGGGAAACATTACC
1-8(♀,+/+)      GGAGCTGGGACTCAGCTGATTGTCATACCTGGTGAGTAGGTTTCTTTCTACTCCAGGGAAACATTACC
1-9(♀,+/+)      GGAGCTGGGACTCAGCTGATTGTCATACCTGGTGAGTAGGTTTCTTTCTACTCCAGGGAAACATTACC
1-10(♀,-/-)     GGAGCTGGGACTCAGCTGATTGTC-TACCTGGTGAGTAGGTTTCTTTCTACTCCAGGGAAACATTACC
1-11(♀,-/-)     GGAGCTGGGACTCAGCTGATTGT----------GAGTAGGTTTCTTTCTACTCCAGGGAAACATTACC
```

(c)

```
WT              GGAGCTGGGACTCAGCTGATTGTCATACCTGGTGAGTAGGTTTCTTTCTACTCCAGGGAAACATTACC
2-1(♂,-/-)      GGAGCTGGGACTCAGCTGATTGTCATACCTGG--------TTTCTTTCTACTCCAGGGAAACATTACC
2-2(♂,-/-)      GGAGCTGGGACTCAGCTGATTGTCATACCTGG----TAGGTTTCTTTCTACTCCAGGGAAACATTACC
2-3(♂,-/-)      GGAGCTGGGACTCAGCTGATTGTCATACCTGG----TAGGTTTCTTTCTACTCCAGGGAAACATTACC
2-4(♂,+/-)      GGAGCTGGGACTCAGCTGATTGTCATACCTGG----TAGGTTTCTTTCTACTCCAGGGAAACATTACC
2-5(♂,+/-)      GGAGCTGGGACTCAGCTGATTGTCATACCTGG----TAGGTTTCTTTCTACTCCAGGGAAACATTACC
2-6(♂,+/-)      GGAGCTGGGACTCAGCTGATTGTCATACCTGGTGAGTAGGTTTCTTTTTCCTCCCGGGGAACATTACC
2-7(♂,+/+)      GGAGCTGGGACTCAGCTGATTGTCATACCTGGTGAGTAGGTTTCTTTCTACTCCAGGGAAACATTACC
2-8(♂,+/+)      GGAGCTGGGACTCAGCTGATTGTCATACCTGGTGAGTAGGTTTCTTTCTACTCCAGGGAAACATTACC
2-9(♂,+/-)      GGAGCTGGGACTCAGCTGATTGTCATACCTGGTGAGGTTCGTTTCTTTCTACGCAAGGAAACATTACC
2-10(♂,+/-)     GGAGCTGGGACTCAGCTGATTGTCATACCTGGTGAGTAGGTTTCTTTCTACTCCAGGGAAACATTACC
2-11(♂,+/-)     GGAGCTGGGACTCAGCTGATTGTCATACCTGG----TAGGTTTCTTTCTACTCCAGGGAAACATTACC
2-12(♀,+/+)     GGAGCTGGGACTCAGCTGATTGTCATACCTGGTGAGGAAGGTTTCTTTCACCCCCGGGAAACCTTTCC
2-13(♀,+/-)     GGAGCTGGGACTCAGCTGATTGTCATACCTGG----TAGGTTTCTTTCTACTCCAGGGAAACATTACC
2-14(♀,+/+)     GGAGCTGGGACTCAGCTGATTGTCATACCTGGTGAGTAGGTTTCTTTCTACTCCAGGGAAACATTACC
2-15(♀,+/+)     GGAGCTGGGACTCAGCTGATTGTCATACCTGGTGAGTAGGTTTCTTTCTACTCCAGGGAAACATTACC
2-16(♀,+/+)     GGAGCTGGGACTCAGCTGATTGTCATACCTGGTGAGTAGGTTTCTTTCTACTCCAGGGAAACATTACC
2-17(♀,+/+)     GGAGCTGGGACTCAGCTGATTGTCATACCTGGTGAGTAGGTTTCTTTCTACTCCAGGGAAACATTACC
```

Fig. S3

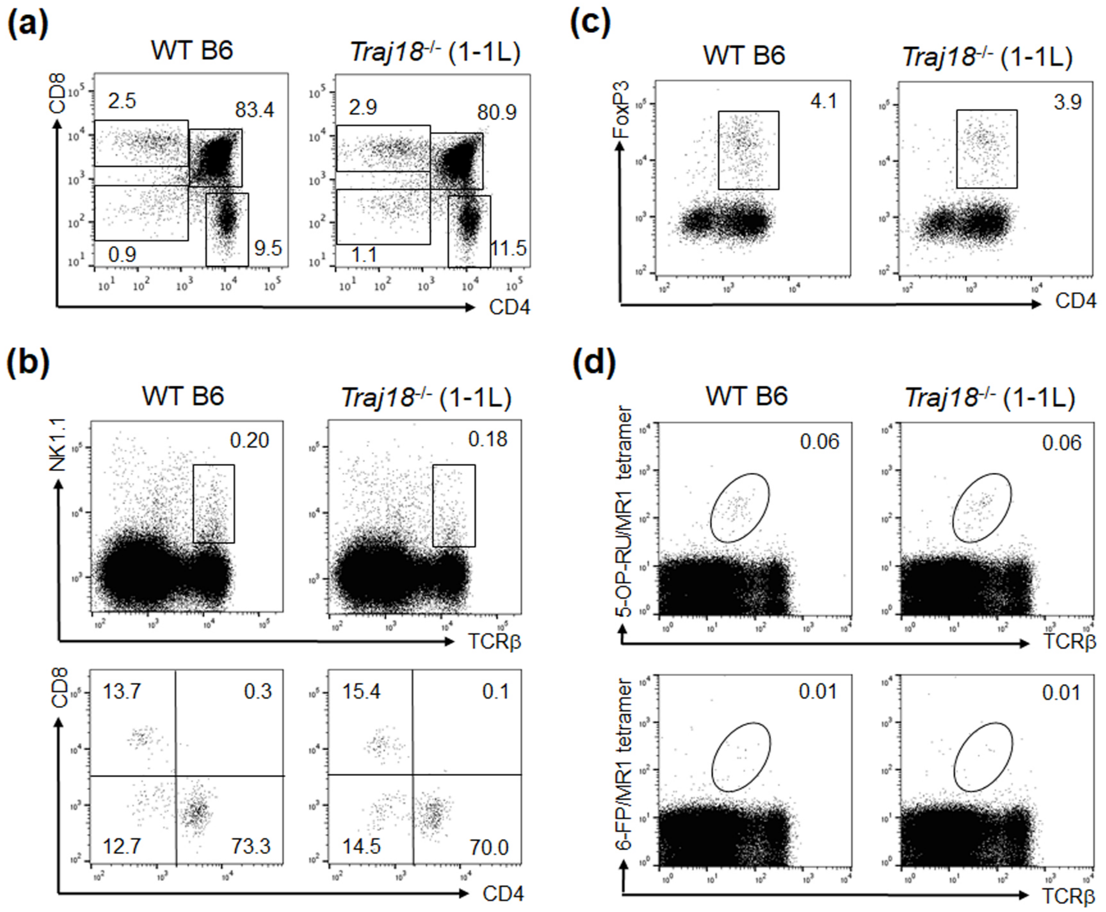
(a)
WT B6
Traj18-/- (1-1L)
CD8
CD4
2.5
83.4
0.9
9.5
2.9
80.9
1.1
11.5
(b)
WT B6
Traj18-/- (1-1L)
NK1.1
TCRβ
0.20
0.18
CD8
CD4
13.7
0.3
12.7
73.3
15.4
0.1
14.5
70.0
(c)
WT B6
Traj18-/- (1-1L)
FoxP3
CD4
4.1
3.9
(d)
WT B6
Traj18-/- (1-1L)
5-OP-RU/MR1 tetramer
TCRβ
0.06
0.06
6-FP/MR1 tetramer
TCRβ
0.01
0.01

Fig.S4

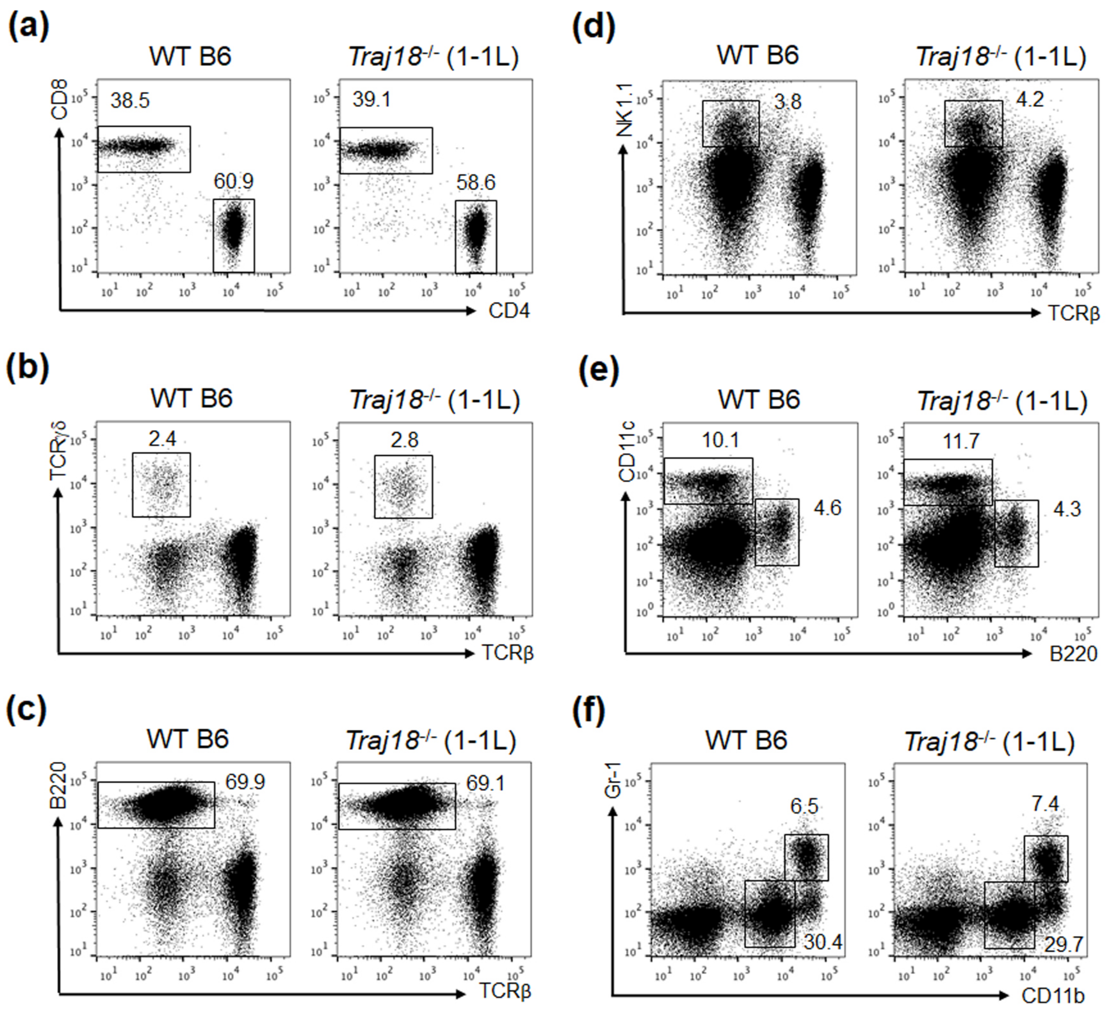
(a)
WT B6
Traj18-/- (1-1L)
CD8
CD4
38.5
60.9
39.1
58.6
(b)
TCRγδ
TCRβ
2.4
2.8
(c)
B220
TCRβ
69.9
69.1
(d)
NK1.1
TCRβ
3.8
4.2
(e)
CD11c
B220
10.1
4.6
11.7
4.3
(f)
Gr-1
CD11b
6.5
30.4
7.4
29.7

Fig.S5

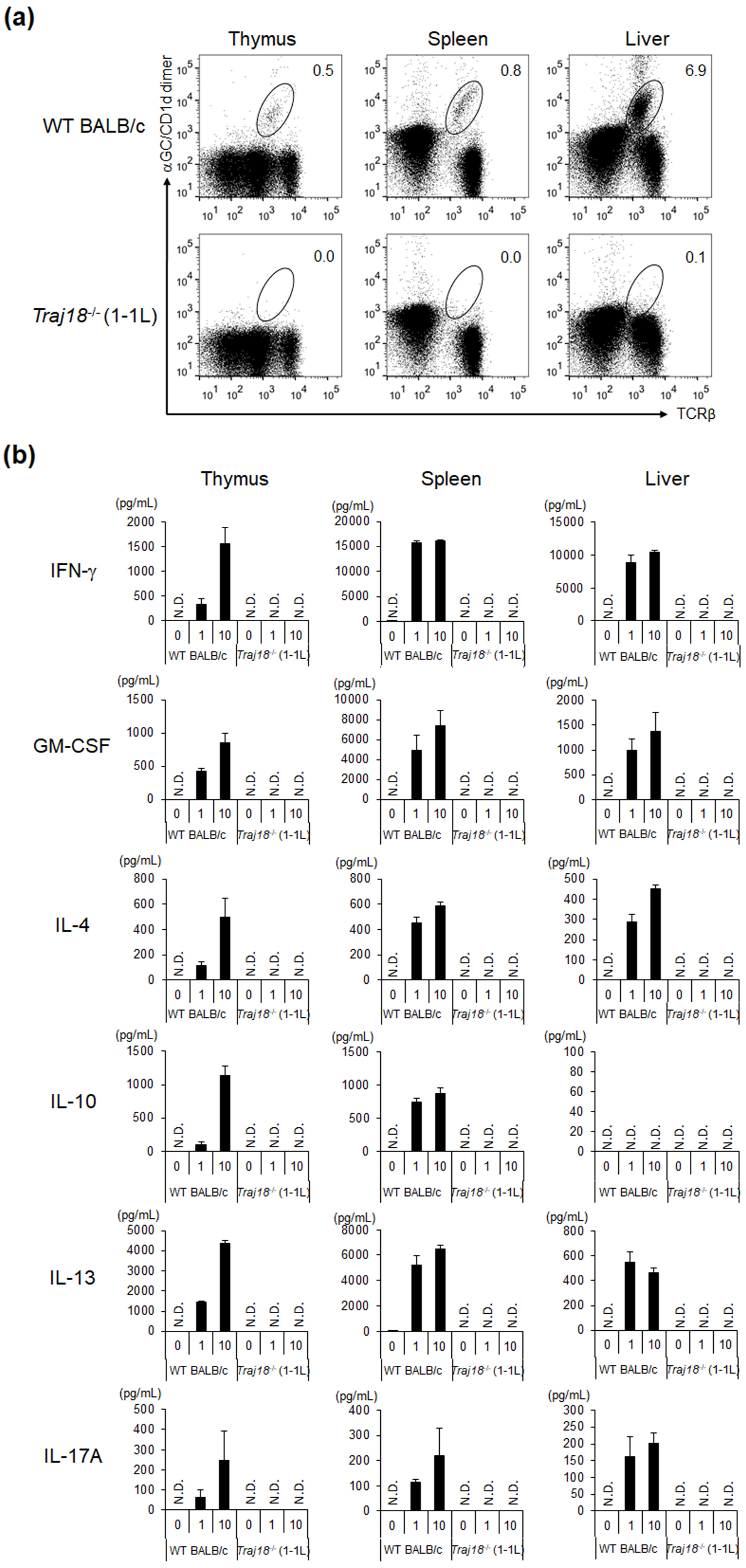
(a)
Thymus
Spleen
Liver
WT BALB/c
Traj18-/- (1-1L)
αGC/CD1d dimer
TCRβ
0.5
0.8
6.9
0.0
0.0
0.1
(b)
Thymus
Spleen
Liver
IFN-γ
GM-CSF
IL-4
IL-10
IL-13
IL-17A
(pg/mL)
N.D.
0
1
10
WT BALB/c
Traj18-/- (1-1L)

Fig. S6

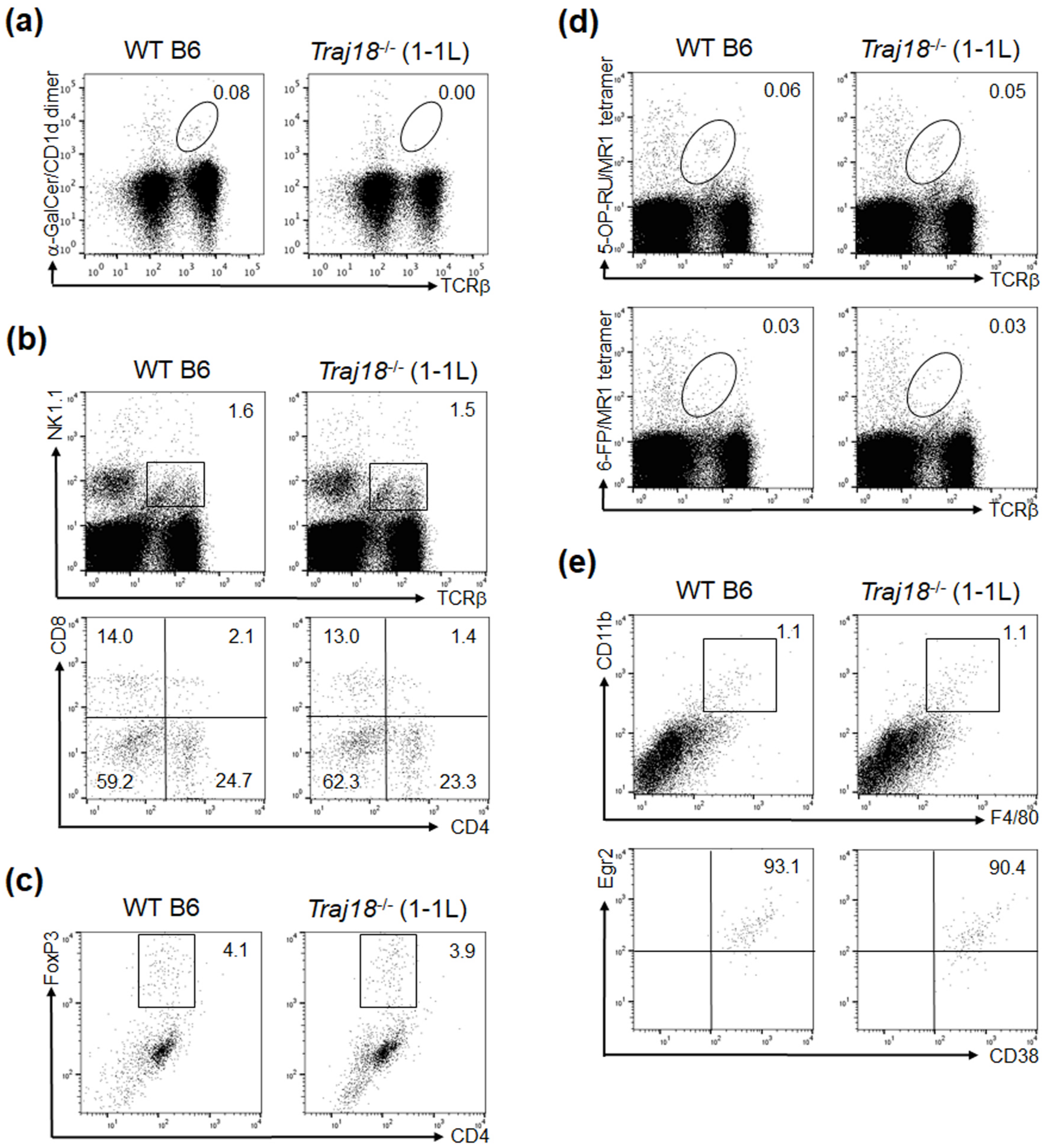

(a)
WT B6
Traj18-/- (1-1L)
α-GalCer/CD1d dimer
0.08
0.00
TCRβ
(b)
WT B6
Traj18-/- (1-1L)
NK1.1
1.6
1.5
TCRβ
CD8
14.0
2.1
59.2
24.7
13.0
1.4
62.3
23.3
CD4
(c)
WT B6
Traj18-/- (1-1L)
FoxP3
4.1
3.9
CD4
(d)
WT B6
Traj18-/- (1-1L)
5-OP-RU/MR1 tetramer
0.06
0.05
TCRβ
6-FP/MR1 tetramer
0.03
0.03
TCRβ
(e)
WT B6
Traj18-/- (1-1L)
CD11b
1.1
1.1
F4/80
Egr2
93.1
90.4
CD38
